# Activity of Proteolytic Enzymes and Level of Cystatin C in the Peripartum Period

**DOI:** 10.1155/2016/7065821

**Published:** 2016-01-20

**Authors:** Anna Cyganek, Aleksandra Wyczalkowska-Tomasik, Patrycja Jarmuzek, Barbara Grzechocinska, Zoulikha Jabiry-Zieniewicz, Leszek Paczek, Miroslaw Wielgos

**Affiliations:** ^1^1st Department of Obstetrics and Gynecology, Medical University of Warsaw, Starynkiewicza 1/3, 02-015 Warsaw, Poland; ^2^Department of Immunology, Transplantology and Internal Diseases, Transplantation Institute, Medical University of Warsaw, Warsaw, Poland

## Abstract

*Objectives*. The aim of the study was to evaluate the activity of cathepsin B, collagenases, trypsin, and plasmin and concentration of cystatin C in serum of healthy pregnant women in peripartum period.* Study Design*. The study group included 45 women in uncomplicated pregnancies. Blood samples were collected in four time points. Enzyme activity was measured by spectrofluorometric method. The level of cystatin C was measured using immunonephelometric method.* Results*. Mean activity of cathepsin B and the level of serum cystatin C were significantly higher in the study group. Collagenase activity was significantly lower in the study group than the control group. No differences in collagenase, plasmin, and trypsin activity on each day of the peripartum period were found.* Conclusion*. High activity of cathepsin B and increased level of cystatin C are typical for women in late pregnancy. Those levels significantly decrease after delivery which can be associated with potential role of those markers in placental separation. The insignificant changes of cystatin C level in the peripartum period seem to exclude the possibility of using cystatin C as a marker for renal insufficiency in the peripartum period but additional research is necessary to investigate the matter further.

## 1. Introduction

Proteolytic enzymes belong to a large group of hydrolases (EC 3). These enzymes are ubiquitous in the human body and are responsible for numerous pivotal functions in both health and disease. Proteolytic enzymes are considered to be important factors in the physiological processes of morphogenesis, cell differentiation, and angiogenesis. Moreover, proteolytic enzymes play a crucial role in cell migration through protein degradation in the extracellular matrix (ECM).

Proteases are classified into two groups: endopeptidases and exopeptidases, depending on the location of peptide bonds that undergo hydrolyses. Based on their catalytic mechanism, proteases are divided into serine (EC 3.4.21), cysteine (EC 3.4.22), aspartate (EC 3.4.22), threonine endopeptidases (EC 3.4.21), and metalloproteases (EC 3.4.24).

The vast majority of studies have evaluated the role of cathepsin B (CTSB) and its inhibitor cystatin C (CYST C) in cellular growth and angiogenesis in cancer diseases. According to recent studies, some cysteine and aspartate proteases are directly involved in the progression and metastasis of colon and ovarian cancer [1, 2].

Cystatin C is considered to be a high-sensitivity marker of renal insufficiency and can also be useful in pregnancy complicated by gestational diabetes mellitus or preeclampsia [3–6].

Both trypsin and metalloproteinases, which belong to the collagenases group, are suggested to be important factors in the progression of cancer disease [7, 8]. Collagenase 1 (MMP 1) and collagenase 3 (MMP 3) tend to accelerate neoplastic progression, whereas collagenase 2 (MMP 8) reduces that process [9–11]. Plasmin, by contrast, regulates blood clotting and the inflammatory response [12].

We found hardly any studies on the role of proteolytic enzymes in pregnancy, labor, and the postpartum period. Based on the literature, proteolytic enzymes are involved in rebuilding of the endometrium and play an important role in the process of implantation and placental development. From the obstetric point of view, hydrolyzing of collagen in the amniotic membranes by MMP 8 can be considered as the underlying pathogenesis of premature rupture of membranes and the development of intrauterine infection [13–16]. Moreover, gestational diabetes mellitus, preeclampsia, and early pregnancy loss can be related to the altered expression of proteolytic enzymes in the trophoblast [17, 18].

Studies on the implantation process and development of the placenta were conducted in a rat model [19, 20]. A few of them analyzed the role of proteolytic enzymes in the involution of the uterus during puerperium [21].

In our study, we evaluated the activity of the most important proteolytic enzymes responsible for extracellular matrix degradation and level of cystatin C in serum of pregnant women in normal peripartum period.

## 2. Material and Methods

### 2.1. Participants

A total of 45 women in uncomplicated pregnancies near the time of delivery, followed up prospectively between October 2012 and April 2013 at the First Department of Obstetrics and Gynecology, Medical University of Warsaw, constituted the study group.

Elective cesarean section was indicated in 26 patients due to the following: previous cesarean section, breech presentation in nulliparous women, previous myomectomy, fetal macrosomia, fetal heart anomaly, cephalopelvic disproportion, or ophthalmologic and orthopedic diseases. Of the entire group, 19 subjects were qualified for preinduction and further induction of labor. The main method of cervical ripening was the intracervical Foley catheter with 60 mL of fluid for 24 hours before labor induction. After performing preinduction and subsequent induction of labor (labor induction, LI), 12 patients delivered vaginally, whereas 7 women delivered by cesarean section. Indications for the surgical procedure included labor dystocia (*n* = 4), fetal distress (*n* = 1), and intrauterine infection (*n* = 2).

Patients were further subdivided into two groups in order to evaluate the influence of delivery mode on the course of the puerperium. The first group included women treated with intracervical catheter who delivered vaginally (VB + LI). The other group included patients after labor preinduction with the Foley catheter that underwent cesarean section (CS + LI).

Patient age in the study group was between 19 and 40 years (mean 31 ± 5). As far as parity is concerned, 19 women were nulliparous and 26 patients (including 13 with a history of cesarean section) were multiparous.

The control group included 10 nonpregnant women, aged between 24 and 39 years (mean 31 ± 5). Blood samples were collected between days 5 and 7 of the menstrual cycle.

Local Bioethics Committee approved the study (number KB/210/2012) and an informed consent was obtained from all participating subjects.

Blood samples in the volume of 2 mL were collected at four time points: 1 day before labor, on the day of labor, and on the first and second days postpartum. The material for the study was venous blood. In order to obtain the serum, blood was centrifuged and the obtained serum was stored at −80°C until being used. In the serum the following were measured: the activity of cathepsin B, collagenases, trypsin, and plasmin and the level of cystatin C.

### 2.2. Analytical Methods

Enzyme activity was measured by spectrofluorometer Perkin Elmer LS-50B (Perkin Elmer, Waltham, USA). Fluorescence measurements were made using the following parameters: wavelength of light induced *λ* = 355 nm and wavelength of light emitted *λ* = 460 nm. Bachem substrates were used in this method (Bachem, Biochemica GmbH, Heidelberg, Germany) [22].

The level of cystatin C was measured using immunonephelometric diagnostic test, N Latex Cystatin C Test (Siemens, Erlangen, Germany). The test was performed on the BN ProSpec Nephelometer (Siemens, Erlangen, Germany), according to the manufacturer's protocol [23].

### 2.3. Statistical Methods

The activity of cathepsin B, trypsin, plasmin, and collagenases (MMP 1, 8, and 13) and the level of cystatin C were evaluated in serum at four time points: 1 day before labor, the day of labor, and the first and second days postpartum. Statistical analysis was performed with STATISTICA 10.0. Due to large intragroup variation, nonparametric Mann-Whitney *U* test was used. Statistical inference was performed by Bonferroni's correction at a significance level of *p* ≤ 0.01.

## 3. Results

No significant complications in the third stage of labor were noted in the entire study group. On the first and second day of the puerperium there were no severe disorders of postpartum uterine involution.

**Figure 1:**
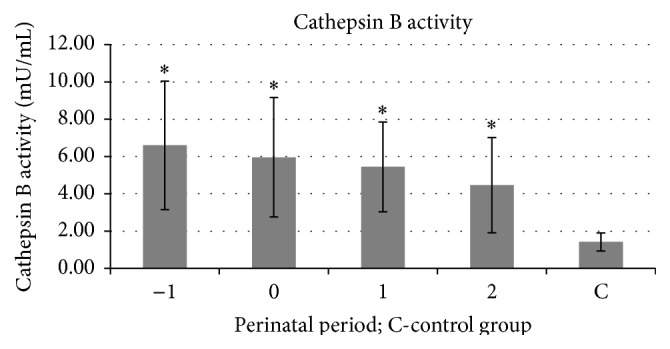
Mean activity of serum cathepsin B 1 day before labor (−1), on the day of labor (0), and on the first (1) and second (2) days postpartum in the study and in the control groups (C); ^*∗*^ *p* ≤ 0.01 statistically significant.

Mean activity of serum cathepsin B in patients from the study group measured 1 day before delivery, on the day of labor, and on the first and second day postpartum was 6.60, 5.96, 5.44, and 4.47 mU/mL, respectively. Mean activity of cathepsin B in the group of pregnant women was statistically significantly higher (*p* < 0.01) as compared to controls, 1.42 mU/mL (Figure 1).

**Figure 2:**
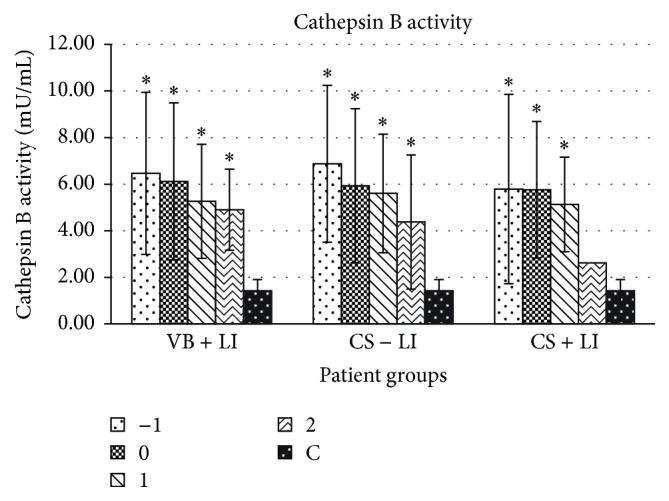
Mean activity of serum cathepsin B 1 day before delivery (−1), on the day of labor (0), and on the first (1) and second (2) days postpartum in three groups of patients as compared to the control group. VB: vaginal birth, CS: cesarean section, LI: labor induction; ^*∗*^ *p* ≤ 0.01 statistically significant.

Mean activity of serum cathepsin B was analyzed in the group of patients after elective cesarean section (CS − LI), patients after vaginal delivery with preinduction and induction of labor (VB + LI), and women after preinduction and induction of labor followed by cesarean section (CS + LI). Cathepsin B activity in all study groups was the highest 1 day before labor (6.46, 6.88, and 5.79 mU/mL, resp.). On the day of delivery, cathepsin B activity in each study group was decreased (6.12, 5.94, and 5.76 mU/mL, resp.). A further, gradual decrease of cathepsin B activity was observed on the first and second day postpartum (5.26, 5.60, and 5.13 mU/mL and 4.90, 4.38, and 2.62 mU/mL, resp.). Generally, mean activity of cathepsin B measured in every patient in the study groups was significantly higher than in controls (C) and amounted to 1.42 mU/mL, *p* ≤ 0.01 (Figure 2, Table 1).

**Figure 3:**
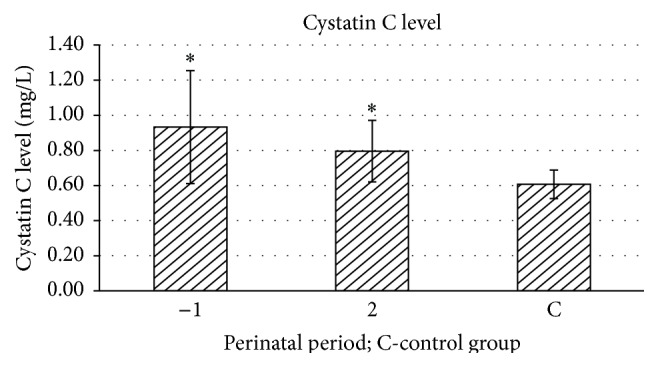
Mean level of cystatin C in the study group 1 day before delivery (−1) and on the second day postpartum (2) and in the control group; ^*∗*^ *p* ≤ 0.01 statistically significant.

Cystatin C level was measured for 4 consecutive days, starting from 1 day before delivery in the study and the control groups. The highest level of serum cystatin C was observed 1 day before labor in every patient, regardless of the delivery mode, and was 0.93 mg/L. On the second day after delivery, mean level of cystatin C was significantly decreased, to 0.80 mg/L. Mean level of cystatin C in the control group was 0.61 mg/L. Gradual decrease of cystatin C level in the next days was associated with the decrease of cathepsin B activity (Figure 3).

Collagenase activity was statistically significantly (*p* < 0.01) increased in the control group as compared to the study group. No differences in collagenase activity on each day of the peripartum period with regard to mode of delivery were noticed (0.61, 0.60, 0.59, and 0.57 mU/mL, resp., and 0.79 mU/mL in control group).

Plasmin activity was comparable in the control and the study groups (3.75, 4.11, 3.76, and 3.48 mU/mL, resp., and 3.34 mU/mL in control group). The results did not show any significant differences among patients from the study group.

In our study, the activity of trypsin did not change in the peripartum period. There were no differences between the study and the control groups (1.58, 1.65, 1.68, and 1.74 mU/mL, resp., and 1.65 mU/mL in control group).

Mean activity of collagenases was inversely proportional to gestational age at delivery. No correlation between patient age, weight, height, parity, and the activity of the investigated enzymes was observed.

## 4. Discussion

In previous studies on animal models, cathepsin B and cystatin C have been indicated as important factors in the process of embryo implantation and formation of the placenta [19, 20]. In pregnant women, cathepsin B is one of the cysteine proteases that shows the highest expression in the first trimester. The disorders of cathepsin B/cystatin C levels correlate with impaired embryo implantation in early pregnancy [24]. According to the literature, an adverse activity of cathepsin B and cystatin C may play an important role in the pathogenesis of recurrent miscarriages [25–27]. In the study of Nakanishi et al., the activity of cystatin C in trophoblast tissue was significantly decreased in a group of women with recurrent miscarriages as compared to women in uncomplicated pregnancies.

In our study, we aimed to evaluate the role of cathepsin B/cystatin C in a group of pregnant women 1 day before delivery, on the day of labor, and on the first two days postpartum. In the recent literature reports, we found no data about the activity of cysteine proteases in serum of pregnant women or placental tissue in the peripartum period.

In our study, the activity of cathepsin B was increased 1 day before delivery. Moreover, mean activity of cathepsin B decreased gradually until the second day postpartum. The changes of cathepsin B activity were strictly correlated to the changes of cystatin C activity. As the correct proportion of cathepsin B and cystatin C is crucial for an uncomplicated implantation and further placentation, an important role of these enzymes in placental abruption may be considered. Proteolytic activity of cathepsin B in the extracellular matrix may be indicated as an important factor of uncomplicated third stage of labor. Our study showed that mode of the delivery has no statistically significant impact on cathepsin B activity changes in serum of pregnant women. Moreover, there were no significant differences in patients who underwent previous cervical ripening and patients without preinduction of labor. No complications of the third stage of labor were noted in any group of patients. High activity of cathepsin B is associated with high level of cystatin C and seems physiologic for an uncomplicated pregnancy in the third trimester. An adequate relation of the enzyme and its inhibitor can be considered as the predictor of uncomplicated third stage of labor.

The highest activity of cathepsin B was observed 1 day before delivery and gradually decreased on the subsequent days of the study. A recent study on a rat model, conducted at the Medical University of Warsaw, yielded comparable results. Mean activity of cathepsin B was measured in serum and uterine tissue. The highest level of cathepsin B activity was observed on the day of labor and was 10 times higher as compared to the control group. The activity of cathepsin B in tissue was 160 times increased as compared to the serum level (study in progress).

The lower activity of cathepsin B in the group of patients with previous cervical ripening may suggest a correlation with uterine contractions in patients after vaginal delivery as well as cesarean section.

The role of cathepsin B and cystatin C in the course of labor and early postpartum remains to be fully elucidated. Activity of cathepsin B was evaluated in immunological disorders and cancer diseases [28]. Cystatin C is believed to play an important protective role as an inhibitor of proteolytic enzymes in cells and tissues in human body.

No significant changes were noted in the activity of collagenases, trypsin, and plasmin throughout the entire course of our study, what may suggest that the investigated enzymes do not play a pivotal role in the peripartum period.

According to the literature, the diagnosis and evaluation of cancer disease progression remain the only recommendation for monitoring cathepsin B activity and the level of cystatin C. Based on our findings, the increased activity of cathepsin B correlated with the increased level of cystatin C can be considered as a predictive factor for preterm delivery or disorders of the third stage of labor.

Guo et al. declared highly elevated levels of cysteine C in women with severe preeclampsia during the perinatal period and they suggested using it as a marker for kidney failure. Taking the results of our study into account it raises doubt over the usage of cysteine C as a marker for kidney failure in normal healthy women during the perinatal period [26, 29].

The changes of cystatin C level in the peripartum period seem to exclude the possibility of using cystatin C as a marker for renal insufficiency but additional research is necessary for investigating the matter further.

## Figures and Tables

**Table 1:** Mean activity of serum cathepsin B 1 day before delivery (−1), on the day of labor (0), and on the first (1) and second (2) days postpartum in three groups of patients; SD: standard deviation; *p*: statistically significant *p* ≤ 0.01.

		−1	0	1	2	C
VB + LI	Mean	6.46	6.12	5.26	4.90	1.42
	SD	3.47	3.37	2.45	1.75	0.49
	*p*	**0.0002**	**0.00009**	**0.0002**	**0.0004**	

CS − LI	Mean	6.88	5.94	5.60	4.38	1.42
	SD	3.37	3.31	2.55	2.88	0.49
	*p*	**0.00002**	**0.00007**	**0.00001**	**0.0002**	

CS + LI	Mean	5.79	5.76	5.13	2.62	1.42
	SD	4.06	2.93	2.03	—	0.49
	*p*	**0.01**	**0.0008**	**0.0008**	—	
